# The Evaluation of Melatonin Effects on Mobilization and Engraftment in Autologous Hematopoietic Stem Cell Transplant Recipients; A Randomized, Double-blind and Placebo-controlled Trial

**DOI:** 10.22037/ijpr.2021.115973.15629

**Published:** 2021

**Authors:** Neda Kazeminia, Mahshid Mehdizadeh, Jamshid Salamzadeh, Sayeh Parkhideh, Farzaneh Dastan, Arash Mahboubi, Maria Tavakoli-Ardakani

**Affiliations:** a *Department of Clinical Pharmacy, School of Pharmacy, Shahid Beheshti University of Medical Sciences, Tehran, Iran. *; b *Hematopoietic Stem Cell Research Center, Shahid Beheshti University of Medical Sciences, Tehran, Iran. *; c *Food Safety Research Center, Shahid Beheshti University of Medical Sciences, Tehran, Iran. *; d *Taleghani Bone Marrow Transplantation Center, Taleghani Hospital, Shahid Beheshti University of Medical Sciences, Tehran, Iran* *.*; e *Pharmaceutical Sciences Research Center, Shahid Beheshti University of Medical Sciences, Tehran, Iran* *.*

**Keywords:** Stem cell, Melatonin, Engraftment, Mobilization, Transplant

## Abstract

Mobilization and engraftment of Hematopoietic Stem Cells (HSCs) are challenging issues in Autologous HSC transplantation (AHSCT) so several attempts such as colony-stimulating factors (CSF) and plerixafor have been used for enhancement of HSCs mobilization and engraftment. In this randomized, double-blind and placebo-controlled study, we evaluated the melatonin’s efficacy and safety, as endogenous CSF inducer, co-administered with Filgrastim in mobilizing and engraftment of HSC. AHSCT patients were randomized to receive either Melatonin or placebo plus filgrastim. Of Fifty-one patients, 26 patients received the melatonin (In mobilization phase 3 mg sublingual twice daily, then 9 mg single dose 30 min before apheresis session and then 3 mg twice daily from +1 until engraftment) and 25 patients received the placebo. The mean number of CD_34_ cells/kg × 10^6 ^in the melatonin group was 6.54 versus 4.22 in the placebo group (*p* = 0.025). The mean day to neutrophil engraftment in the melatonin group was 11.69 ± 2.093, whereas 12.68 ± 2.42 days in the placebo group (*p* = 0.021). In this study, the second apheresis session requirement, the use of plerixafor and hospital stay duration, were comparable between the two groups. Considering the result of the study, it could be suggested that melatonin plus Filgrastim can be effectively used in AHSCT patients to enhance the number of peripheral CD_34_ cells/kg × 10^6^ and decrease the day number of neutrophil engraftment.

## Introduction

Hematopoietic stem-cell transplantation (HSCT) is a probable curative therapy used for various hematopoietic malignancies (leukemias, lymphomas, and myeloma) and several hematologic and immune disorders (*e.g*., hemophagocytic disorders, myelodysplasia, and innate immune defects) (1). Moreover, this can be done as autologous, allogeneic or syngeneic (2).

In autologous HSCT (AHSCT), HSCs can be collected from the Peripheral Blood Stem Cells (PBSCs) or the Bone Marrow (BM) (3, 4). Nowadays, PBSCs strategy due to the accelerated reconstitution has become a preferred source and the most widely used practical therapeutic approach compared to BM (5).

However, obtaining sufficient CD_34_^+^ cells for AHSCT persists as one of the challenges in PBSCs collection strategy. So, due to this reason, approximately 5% to 40% of patients intended for AHSCT are unable to achieve adequate CD_34_^+^ cells/kg and to define Poor Mobilizer (PM) (6, 7). PM patients require multiple mobilization attempts, and in this regard, their quality of life, engraftment (8), transplantation outcomes, and healthcare costs are affected by the PM as a major factor (9). Several clinical trials have previously investigated different stem cell mobilization strategies such as granulocyte-macrophage colony-stimulating factor (GM-CSF), G-CSF, and C-X-C chemokine receptor type 4 (CXCR4) inhibition with plerixafor or recovery from cytotoxic chemotherapy, to increase circulating of CD_34_^+^ hematopoietic stem and progenitor cells (10-12).

Melatonin, as a neurohormonal peptide produced by the pineal gland, can improve several physiological functions; for instance, the orientation of circadian rhythm, reproduction, mood and immune function (13). In addition, it has anti-inflammatory (14) and anti-oxidative properties (15). As well, melatonin plays regulatory roles in the differentiation, proliferation, and migration of stem cells such as Neural Stem Cells (NSCs) (16), Hemopoietic Stem Cells (17), Spermatogonial Stem Cells (18), Embryonic Stem Cells (14,19) and Pluripotent Stem Cells (iPSC) (20). *In-vivo *and* in-vitro *studies have reported that melatonin, as the regulator of interleukin 4, can induce the release of endogenous GM-CSF by adherent stromal cells stimulation (13). Moreover, this agent has a hematopoietic rescue effect as well as some antitumoral and immune-enhancing properties due to acting in an endocrine-cytokine mechanism (21). 

Up to now, there is no clinical trial for the administration of melatonin as a GM-CSF inducer agent for improvement of mobilization and engraftment in AHSCT recipients. This double-blind, placebo-controlled clinical trial was designed to evaluate the efficacy and safety of melatonin compared to the placebo in mobilizing CD_34_^+^ cells and time to engraftment in patients with AHSCT, as the primary endpoints. Additionally, some parameters like the use of plerixafor, need the second apheresis, so the G-CSF dose requirement in mobilization and engraftment phases, duration of grade II neutropenia and hospital stay, Insomnia Severity Index (ISI), and Hospital Anxiety and Depression Scale (HADS) were investigated as the secondary outcomes. 

## Experimental

*Study design and study population*

This was a randomized, double-blind, placebo-controlled study conducted to evaluate the effectiveness of melatonin plus G-CSF in both HSC mobilization and engraftment in patients undergoing AHSCT. For this purpose, 93 patients aged between 18 and 70 years old who were eligible for AHSCT, enrolled in this study from October 2019, to December 2020. The study was approved by the Ethics Committee of School of Medicine – Shahid Beheshti University of Medical Sciences (IR.SBMU.PHARMACY.REC.1398.214) in September 2019, and also registered in the Iranian Registry of Clinical Trial under the following code IRCT20100127003210N20. The inclusion criteria of this study were as follows: 1) the patients aged between 18 and 70 years old diagnosed with hematopoietic malignancy and meeting the eligibility for autologous hematopoietic stem cell transplantation; 2) the first transplantation, 3) the first mobilization attempt; 4) adequate liver (AST and ALT ≤ 3 upper limit normal) and kidney functions (creatinine ≤ 2 mg/dL), and 5) no plerixafor administration planning at first. Moreover, the exclusion criteria were as follows: 1) inability to use sublingual tablets, 2) regular use of ≥ two antidepressants, 3) receiving hypnotic and sedative medicines for more than one week, 4) taking medicines with melatonin major interactions with such as fluvoxamine, cimetidine, and benzodiazepines (alprazolam, chlordiazepoxide, clonazepam, lorazepam, and oxazepam). 

*Intervention*

The patients were randomized 1:1 using the block randomization method, to receive either melatonin (Sublingual tablet 3 mg, Vana Darou Gostar company) or placebo (Sublingual tablet, manufactured by the pharmaceutical department of Shahid Beheshti University of Medical Sciences) and then underwent mobilization with G-CSF according to the local practice guidelines as 10-16 µg/kg/day for mobilization and 5-10 µg/kg/day from day +5 for the engraftment phase. The physicians, nurses, data analysts, and patients were blinded to all the study and analysis processes. On the first mobilization day, the patients were given either melatonin or placebo 3 mg Sublingual tablets twice daily and 9 mg SL 30 min were administered before the first apheresis session for achieving the optimal target range 3-5 × 10^6^ CD_34_^+^ cells/kg. After the first apheresis session for poor stem cell mobilizing patients (< 3× 10^6^ CD_34_^+^ cells/kg) the second apheresis session was done with/without plerixafor administration. After reaching the CD_34_^+^ cells/kg target, melatonin or placebo 3 mg SL twice daily were administered from +1 after transplantation until engraftment occurred. 

*Primary and secondary endpoints*

The primary endpoints were the number of peripheral bloods CD_34_^+^ cell/kg × 10^6^ and the number of days to neutrophil engraftment. However, the secondary endpoints were considered as the use of plerixafor, the second apheresis requirement, duration of both neutropenia and hospitalization, the assessment of insomnia/sleep disturbance, and signs and symptoms of anxiety and depression according to ISI and HADS questionnaire respectively. The HADS is a 14 items self-assessment questionnaire for evaluation of anxiety and depression in hospitalized patients. Seven items for each subscale of depression and anxiety and each item have 4-point (0-3). A higher total score shows a greater level of anxiety and depression. Also, ISI is a 7-item self-rating form that demonstrates the nature, severity, and effect of insomnia. A total score extending from 0 to 28. The total score is defined as follows: absence of insomnia (0–7); sub-threshold insomnia (8–14); moderate insomnia (15–21); and severe insomnia (22–28).

 Neutrophil engraftment and grade II neutropenia were usually defined as an absolute neutrophil count (ANC) of ≥0.5 × 10^9^/L for 3 consecutive days with no transfusion and neutropenia grade II ≥1,000- 1,500/mm^3^, respectively. Thereafter, ISI and HADS were evaluated firstly at baseline and then at weekly follow-ups until the time that engraftment occurred. 

*Statistical analysis*

The number of CD_34_^+^ (1 × 10^6 ^cell/kg ± 2.22) and PMN engraftment days (1 day ± 1.57) as primary endpoints were considered for calculating sample size. The final sample size has been calculated as 24 patients per group, to provide a power of 80%, alpha error of 5%, and exception of 20% drop-out rate equally from the two studied groups. The obtained data were then analyzed by the IBM SPSS® software platform version 26. The t-test was used to compare the CD_34_^+^ cell/kg × 10^6 ^means, as well as the number of days of both neutropenia and hospital stay duration whereas GCSF mobilization/engraftment dose (µg/kg/day) and engraftment duration (day) were calculated by the Mann–Whitney U test. The chi-squared test was also used to analyze the Second apheresis requirement and the use of plerixafor. Finally, the ISI and HADS were analyzed by t-test for each group separately during follow-up weeks and by repeated measure ANOVA test for comparison between two groups. 

## Results

*Patients Characteristics*

**Figure 1:**
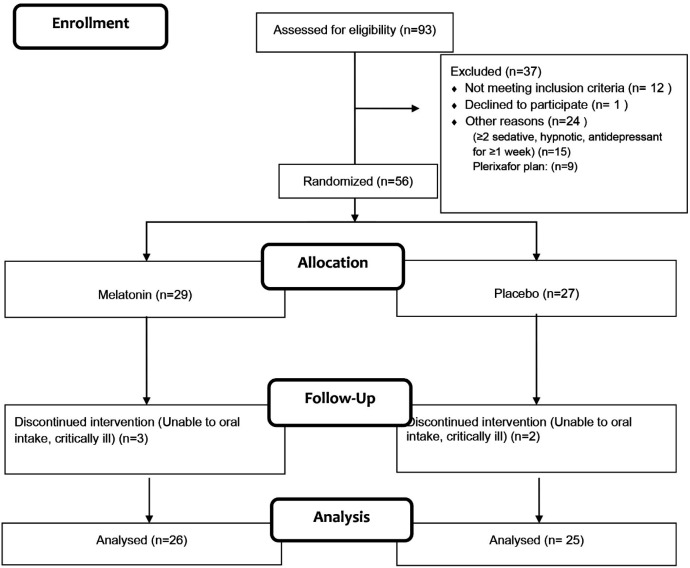
Study Flowchart

Of 93 patients enrolled in this study, 56 (61%) subjects were randomized after meeting the inclusion criteria (Figure 1). Finally, 51 patients finished the study. The baseline demographics and clinical characteristics of the included patients were comparable between the two groups (Table 1).

*Efficacy*

*Mobilization phase*

In the mobilization phase (Table 2), a significantly greater mean of peripheral blood CD_34_^+^ cells/kg was reported in the melatonin group compared to the placebo group (*p* = 0.025). On the other hand, the patients receiving plerixafor to achieve the primary endpoint of 3-5 × 10^6^ CD_34_^+^ cells/kg showed no significant difference compared to the other group (*p* = 0.541). Similarly, the second apheresis session requirement was evaluated between the two groups but the difference was not statistically significant (0.555). The median G-CSF dose (µg/kg/day) of the mobilization phase was comparable between the two groups (*p* = 0.972).

*Transplantation and engraftment phases*

In the engraftment phase (Table 2), the comparisons between the meantime to neutrophil engraftment in the melatonin and placebo groups showed a statistically significant difference (*p* = 0.021). No patient was documented as having the experience of engraftment failure either in the melatonin group or in the placebo group. The duration of grade II neutropenia was observed to be significantly lower in the melatonin group compared with the placebo group (*p* = 0.033). As well, the G-CSF dose in the engraftment phase was comparable between the two groups (*p* = 0.373).

*Hospital stay duration*

The mean of hospitalization days in the melatonin-treated group was comparable with the placebo-treated group and no statistically significant difference was found between these two groups (*p* = 0.111) (Table 2).

*Adverse effect*

In this study, no serious adverse effects were observed among the participants of the two groups. However, in the melatonin group, two cases of dyspepsia and three cases of inability of sublingual use were reported. Accordingly, this adverse effect was related to different menthol contents and sources of sublingual melatonin tablets as flavors in new batches.

*ISI and HADS*

In this study, quality of sleep and anxiety scores were evaluated by ISI and HADS questionnaire firstly at baseline and then at weekly follow-ups for two weeks. During hospitalization, according to ISI and HADS scores, there was no statistically significant difference between the melatonin and placebo-treated groups. from a different perspective, in melatonin and placebo groups the ISI score improved between the second and third weeks but only in the melatonin group, the ISI score had improved between the first and second week. For the HADS score melatonin group had statistically significant improvement after the second and during the second to the third week as shown in Table 3. 

## Discussion

To date, there is no report of melatonin as a mobilizing agent in patients undergoing AHSCT, but several animal studies have reported the regulatory role of melatonin in viability, proliferation, and differentiation of stem cells by having anti-inflammatory, anti-oxidant, and anti-apoptosis properties (21)(13, 22). Maestroni *et al.* in their study reported that melatonin with neuroendocrine cytokine mechanism can rescue bone marrow cells from apoptosis of cancer chemotherapy compounds either under *in-vivo* or under *in-vitro* condition, by regulation of interleukin 4 release from bone marrow T-helper cells as well as the stimulation of stromal cell to produce GM-CSF subsequently (22). Consistent with several previously performed animal studies, Xin Yu et al. have also reported the protective role of melatonin in neural stem cells (NSC) as immature precursors of the central nervous system. It was indicated that melatonin could regulate proliferation, differentiation, and survival of NSC by the neuroimmune-endocrine axis and the inhibition of interleukin-18 in the neurogenesis process of many neurological disorders such as Parkinson, Alzheimer, and ischemic brain injury (23).

To the best of our knowledge, this article was the first study investigating the efficacy and safety of melatonin on improving PBSC mobilization and engraftment of HSCT. As well, several studies have previously shown the efficacy and safety of plerixafor as a CXCR4 receptor antagonist on HSC mobilization and collection. In the two-phase III studies, Micallef *et al. *demonstrated the efficacy and safety of plerixafor for improving HSC mobilization and collection among Non-Hodgkin Lymphoma (NHL) or Multiple Myeloma patients who underwent AHSCT (1,18). This study reported that plerixafor plus G-CSF compared with placebo plus G-CSF can increase the proportion of older patients significantly who achieved ≥ 2 × 10^6^ CD_34_^+^ cells/kg within 4 days of apheresis and versus those patients treated by placebo + G-CSF (NHL: 50.9 *vs.* 25.4%, *p* < 0.001; MM: 69.6 *vs.* 23.7%, *p* < 0.001). However, the median times to neutrophil and platelet engraftment were comparable between these two groups. In this phase 2, randomized, double-blinded, placebo-controlled study, a significantly higher mean of CD_34_^+^ cells/kg was achieved in patients receiving melatonin plus G-CSF compared with those receiving placebo plus G-CSF (*p* ˂ 0.05). One of the most important findings of this study was the neutrophil engraftment as well as the neutropenia duration. Accordingly, these were significantly shorter in the melatonin plus G-CSF compared to the Placebo plus G-CSF (*p* = 0.021, 0.033 respectively). Regardless of the mobilization agent received, each patient who underwent AHSCT neither melatonin nor placebo-treated, attained successful neutrophil engraftment. Of note, the adverse events reported in the melatonin group were as follows: dyspepsia (2%) and inability to sublingual tablet consumption in three patients (11%). The defined batch number of melatonin recalls to the company and the patients did not continue the study. Furthermore, no unexpected adverse effects were observed in the patients of the placebo group.

Altogether, the analysis of our data demonstrated the need for plerixafor administration and additional apheresis sessions, hospital stay duration, and G-CSF dose in both the mobilization and engraftment phases, which were not significantly different in both groups. However, the patients who received melatonin had shorter hospital stay duration and lower G-CSF dose in the mobilization and engraftment phases. Sleep disturbance, insomnia, and anxiety are known as problematic issues among HSCT candidates. Although literature on sleep and anxiety prevalence among patients intended to HSCT are limited, many studies have found sleep disturbance (32-50%), subthreshold (48%), moderate (23%), and severe (3%) levels of insomnia and anxiety, which are highly prevalent in these patients prior to transplant, during, and post-transplant (41%) periods. According to the previous studies, it can be said that melatonin, as a sleep inducer hormone, can improve quality of sleep and insomnia and decrease anxiety by balancing between GABAergic and glutamatergic transmissions.

The result of the presented study (Table 3) showed that in each group separately, although improvement in the quality of sleep was observed in the second week in both groups, melatonin has a positive effect in the first week. Furthermore, melatonin had a statistically significant effect on the stress and anxiety of patients after the first week. In conclusion, it seems that the anti-anxiety and anti-depression effects of melatonin were demonstrated in the second week while the positive effect of melatonin on quality of sleep was seen within the first week and mobilization phase in AHSCT patients. From the other perspective, the statistical analysis in both groups showed that insomnia and anxiety improvement according to ISI and HADS questionnaires were comparable between the two groups of melatonin and placebo with no statistically significant difference. It seems that medical-related distress such as adverse effects of chemotherapy, long-term hospitalization, and fear of recurrence are effective variables that decrease the impact of melatonin

**Table 1:** Demographic and clinical characteristics of AHSCT patients

		Melatonin (n = 26)	Placebo (n = 25)	*P-value*
Baseline demographic characteristics				
Age, years old (Mean ± SD)	42.19 ± 13.20		40.44 ± 15.61	0.667
Female n (%)	11(42.30)		13(52)	0.579
**Baseline clinical characteristics**				
PDH n (%**)**	6 (23.07)		5(20.00)	1.000
PMH n (%)	5(19.23)		4(16)	1.000
Allergic Hx n (%)	3(11.53)		1(4)	0.610
Malignancy Type (LYM/MM/AML) n (%)	16/9/1 (64/34.61/3.84)		15/9/1 (60/36/4)	0.994
**Chemotherapy and radiotherapy statuses **				
>1 chemotherapy cycle (%)	9 (34.61)		7 (28.00)	1.000
Prior Radiotherapy n (%)	8 (30.76)		11(44)	0.393
Prior Heavily treated n (%)	13(50)		16(64)	0.400
Heavily treated type (RT/Cycle/RT + Cycle)^ ¶ ^n (%)	4/5/4 (15.38/19.23/15.38)		8/4/4(32/16/16)	0.597

PDH: Past Drug History; PMH: Past Medical History; Hx: History; LYM: Lymphoma; MM: Multiple Myeloma; AML: Acute Myeloid Lymphoma; RT: Radiotherapy; ^¶^Cycle: more than one chemotherapy cycle during the disease.

**Table 2:** The result of study during mobilization and engraftment phase

	Melatonin (n = 26)	Placebo (n = 25)	*P-value*
Mobilization phase			
Peripheral blood × 10^6 ^CD_34_^+^ cells/kg (Mean ± SD)	6.54 (± 4.454)	4.22 (± 2.3)	0.025
Receiving plerixafor (n (%))	6 (23.07)	8 (32)	0.541
The second apheresis session requirement (n (%))	7 (26.92)	9(36)	0.555
The Median G-CSF dose (µg/kg/day) (Median (IQR))	9.33 (4.608)	9.34 (4.235)	0.972
**Transplantation and engraftment phases**			
The mean time (days) to neutrophil engraftment (Mean ± SD)	11.69 ± 2.093	12.68 ± 2.42	0.021
The duration (days) of grade II neutropenia (Mean ± SD)	8.73 ± 2.50	9.29 ± 3.11	0.033
The G-CSF dose in the engraftment phase (µg/kg/day) (Mean ± SD)	5.038 ± 2.366	5.57 ± 1.804	0.373
**Hospital stay duration**			
The mean of hospitalization days (Mean ± SD)	21.27 ± 2.80	22.68 ± 3.38	0.111

G-CSF: Granulocyte colony-stimulating factor; IQR: interquartile range; SD: standard deviation.

**Table 3:** ^A^The evaluation of melatonin and placebo efficacy ISI and HADS score. ^B^The evaluation of melatonin and placebo efficacy on ISI and HADS score during follow up weeks

Dependent variable	*p-*value	
Treatment effect on ISI and HADS score between two groups ^A^		
**ISI**	0.39	
**HADS** ^1^	0.45	
Comparisons of ISI and HADS score during follow up weeks ^B^		
**follow up weeks**	Melatonin	Placebo
**ISI w1- ISI w2**	0.009	0.529
**ISI w1 – ISI w3**	0.766	0.364
**ISI w2 -ISI w3**	0.008	0.026
**HADS w1- HADS w2**	0.281	0.144
**HADS w1 – HADS w3**	0.010	0.521
**HADS w2 - HADS w3**	0.008	0.845

ISI: Insomnia Severity Index; HADS: Hospital Anxiety and Depression Scale; w1: first week; w2: second week; w3: third week.

## Conclusion

Successful mobilization of stem cells due to costs of mobilization, mobilizing agent’s adverse effects, and clinical outcomes, is mainly challenging among HSCT patients. Based on our data, the upfront use of melatonin could be considered as a safe, easily available, and well-tolerated option for stem cell mobilization and engraftment along with G-CSF among patients intended for AHSCT. Melatonin administration appears to be associated with a significant increase in both the PBSC collection and CD_34_^+^ cell mean probability, as well as providing fewer engraftment days. This encouraging information along with the following studies will permit researchers to define clinical algorithms to PBSCs mobilization appropriately in patients who are candidates for AHSCT in their clinical settings.

## Conflict of Interest

The authors certify that they have NO affiliations with or involvement in any organization or entity with any financial interest (such as honoraria; educational grants; participation in speakers’ bureaus; membership, employment, consultancies, stock ownership, or other equity interest; and expert testimony or patent-licensing arrangements), or non-financial interest (such as personal or professional relationships, affiliations, knowledge or beliefs) in the subject matter or materials discussed in this manuscript.
